# Diagnostic and Prognostic Role of Serum Interleukin-6 in Malignant Transformation of Liver Cirrhosis

**DOI:** 10.5005/jp-journals-10018-1253

**Published:** 2018-05-01

**Authors:** Mustafa Yakut, Hasan Özkan, Muhammed F Karakaya, Harun Erdal

**Affiliations:** 1 Department of Gastroenterology, Memorial Diyarbakir Hospital, Diyarbakir, Turkey; 2 Department of Gastroenterology, Ankara University School of Medicine, Ankara, Turkey; 3 Department of Gastroenterology, Düzce Public Hospital, Düzce, Turkey

**Keywords:** Cirrhosis, Diagnosis, Hepatocellular carcinoma, Interleukin-6, Prognostic role.

## Abstract

**Aim:**

Alpha-fetoprotein (AFP) is still the most commonly used and the single most recommended marker in the diagnosis of hepatocellular carcinoma (HCC). Interleukin (IL)-6 is a circular cytokine and its role on carcinogenesis in various hematological and solid tumors is clearly documented. A combination of serum IL-6 and AFP may provide beneficial information regarding early diagnosis of HCC. In this study, the effect of plasma IL-6 level in the diagnosis of HCC was investigated.

**Materials and methods:**

A total of 130 patients with liver cirrhosis, together with 30 control cases were enrolled in the trial. A diagnosis of HCC was present in 75 patients (57.6%) in the liver cirrhosis group. Blood samples were obtained from the enrolled study and control cases. Alpha-fetoprotein was quantified by chemiluminescent method. Plasma IL-6 levels of samples obtained at -80°C were quantified by human IL-6 BMS213/2 BMS213/2TEN kit.

**Results:**

The HCC patients were older than the patients in the cirrhosis group (p = 0.016). On comparison of the HCC patients with the control group, AFP (p < 0.001) and IL-6 (p < 0.001) were significantly higher among the HCC patients. Comparison of HCC patients with liver cirrhosis cases with no diagnosis of HCC revealed significantly high AFP (p < 0.001) and IL-6 levels (p < 0.001) in HCC group. Cutoff value for IL-6 was calculated as 5.73 (pg/mL). No difference was detected in AFP (p = 0.600) and IL-6 (0.344) in all three subgroups. A total of 17 patients died during a mean follow-up period of 32.9 months. No correlation was found between mean AFP values and IL-6 values and survival rates.

**Conclusion:**

Plasma IL-6 level was found to be significant in the diagnosis of HCC. Alpha-fetoprotein and IL-6 provided no advantage in terms of early diagnosis of HCC and no correlation was observed between these markers and survival.

**How to cite this article:** Yakut M, Özkan H, Karakaya MF, Erdal H. Diagnostic and Prognostic Role of Serum Interleukin-6 in Malignant Transformation of Liver Cirrhosis. Euroasian J Hepato-Gastroenterol 2018;8(1):23-30.

## INTRODUCTION

Hepatocellular carcinoma is the most common primary liver tumor worldwide. Hepatocellular carcinoma usually develops based on cirrhotic liver and it still presents a poor prognosis.^1-3^ The prognosis of HCC is strongly correlated with early diagnosis. Currently, diagnosis is based on imaging techniques and detection of high AFP levels.^4,5^ For an early diagnosis of HCC, regular screening programs comprising imaging techniques and serum tumor markers should be implemented in populations at risk. For the time being, AFP is still the most commonly used and the single most recommended marker in the diagnosis of HCC.^6^ A number of tumor markers were investigated in the early diagnosis of HCC.

Interleukin-6 is a pleiotropic cytokine. During inflammation, IL-6 regulates the response of certain liver-specific transcription factors.^7^ In addition, IL-6 is a positive regulator of growth, and IL-1, IL-3, and IL-6 play a role in the proliferation, differentiation, and apoptosis of myeloid cells.^8^ The role of this proinflammatory cytokine in the activation and differentiation of cytotoxic T cells and NK cells is well documented; based on this activity, IL-6 pathway is being used in cancer treatment.^8,9^ The IL-6 is a circular cytokine and its role on carcinogenesis in various hematological and solid tumors is clearly documented. It inhibits apoptosis in cancer cells and stimulates angiogenesis. It regulates growth in solid tumors through paracrine and autocrine actions. Levels of IL-6 play a role in proliferation of cancer cells in solid tumors. Also, IL-6 is associated with the stage of tumor and survival.^8^ Cirrhotic patients are chronically exposed to cytokines, in particular to IL-6. Hepatitis B X-protein upregulates nuclear factor (NF)-kB and IL-6 levels.^7^ A combination of serum IL-6 and AFP may provide beneficial information regarding early diagnosis of HCC.

In the current trial, the effect of plasma IL-6 level on the diagnosis of HCC was investigated. In addition, the efficiency of this marker in early stages of HCC and its correlation with survival in this disease were also evaluated.

## MATERIALS AND METHODS

### Patients

A total of 130 patients with liver cirrhosis, together with 30 control cases enrolled in the trial, were followed-up in the Gastroenterology Department of Ankara University from February to September 2016. Over a period of 32.9 months, a total of 130 patients with cirrhosis were included in the analysis.

A diagnosis of HCC was present in 75 patients (57.6%) in the liver cirrhosis group. All patients were followed-up every 3 to 6 months. Basic demographic data for all patients were recorded. The etiology of the disease was investigated in all patients; Child and model for end-stage liver disease scores were calculated. Exitus cases during follow-up were recorded. Ethical Committee approval was completed prior to initiation of the trial.

### Measurement of Plasma AFP and IL-6 Levels

Blood samples obtained from enrolled study and control cases were centrifuged for 7 minutes at a speed of 5,000 cycles and plasma was separated. Plasma samples were stored in deep freeze at -80°C. Alpha-fetoprotein was quantified by chemiluminescent method. An upper limit of our laboratory, 13 ng/mL, was accepted as the cutoff value. Plasma IL-6 levels of samples obtained at -80°C were quantified by human IL-6 BMS213/2 BMS213/2TEN kit, Bender Med Systems GmbH Campus Vienna Bio Center 2; 1030, Wien (Austria).

### Staging and Classification into Subgroups in HCC Patients

Patients in the HCC group were evaluated according to the presence of extrahepatic metastasis. Cases in the HCC group were also investigated in terms of portal vein thrombosis.

All HCC patients were categorized by tumor, node, and metastasis (TNM) classification. Tumor markers of AFP and plasma IL-6, presence of extrahepatic metastasis, presence of portal vein thrombosis, and Child scores of all HCC patients were compared according to TNM classification. Patients were divided into three subgroups in accordance with Milan criteria.^10^ Alpha-fetoprotein and plasma IL-6 levels in all HCC patients were compared in terms of these three subgroups and significance of tumor markers were investigated in small-sized tumors.

### Statistical Analysis

Statistical Package for the Social Sciences 16.00 program was used in data analysis. Comparison of nonparametric values between the groups was realized by chi-square test. For comparison of means between the groups, Student’s t-test was used in cases where there were two groups and variance was normal; for comparison of two groups with abnormal variance, Mann-Whitney U-test was utilized. For comparison of groups >2, analysis of variance (ANOVA) was used for normal variance and Kruskal-Wallis test was used for abnormal variance. In cases where p-value was calculated as significant, based on test results, multiple comparative tests were used to determine the group from which the variance originated. p < 0.05 was regarded as statistically significant. Accuracy, sensitivity, and specificity of AFP and IL-6, positive predictive value (PPV), negative predictive value (NPV), and Youden’s index J (where J = sensitivity + specificity - 1) were calculated. Receiver operating characteristic (ROC) curves and the respective areas under the curves (AUCs) were calculated. Significance value was accepted as p < 0.05. Correlation between variables was performed by Spearman rank test. Correlation of AFP and IL-6 with survival was evaluated by Kaplan-Meier survival curve and Cox regression analysis.

## RESULTS

### Baseline Demographic, Clinical and Laboratory Characteristics of HCC, Cirrhosis, and Healthy Control Groups

In the current trial, 30 healthy control cases and 130 patients with liver cirrhosis were investigated. Among 130 patients with liver cirrhosis evaluated in our trial, HCC was not determined in 55 cases. On the contrary, a diagnosis of HCC was detected in 75 patients. A comparison of HCC patients with the healthy control group yielded no difference between the groups in terms of age (p = 0.076). The mean age in the control group was determined as 65.21, standard deviation (SD) 12.1, the median 70 (35-81), while mean age in HCC group was 62.8, SD 9.9, and the median 63 (40-85). In the control group, 18 individuals were men (60%) and 22 were women (40%). In the HCC group, 54 patients were women (72%) and 21 were men (28%). No difference was found between the HCC and control groups in terms of gender (p = 0.231). In basal evaluations, values of aspartate transaminase (AST), alanine transaminase (ALT), the total bilirubin, and direct bilirubin in the HCC group were significantly higher than the control group (Table 1).

The HCC patients were older than the patients in the cirrhosis group (p = 0.016). While the mean age in the HCC group was 62.8, SD 9.9, the median 63 (40-85), and the corresponding values in the cirrhosis group was 57.4, SD 12.7 and the median 58 (28-85). No difference was found between the two groups in terms of mean ALT, total bilirubin, direct bilirubin, creatinine, albumin, and international normalized ratio (INR). The mean AST value was higher in the HCC group (mean 99.9, SD 138.5 *vs* mean 63.8, SD 80; p = 0.016). Evaluation of the two groups in terms of basal Child-Pugh scores revealed higher in the HCC group (p = 0.022; Table 1).

### Comparison of AFP and Plasma IL-6 Levels in Healthy Control Group, Cirrhosis Patients, and HCC Patients

**Graphs 1A and B:**
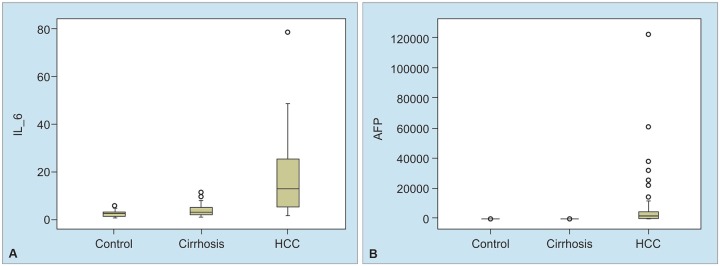
Box-plot graphs of AFP and IL-6 values in control group, cirrhotic patients, and HCC patients

Upon comparison of HCC (75 patients) in the control group (n = 30), AFP (p < 0.001) and IL-6 (p < 0.001) were significantly upper among HCC patients (Table 2 and Graph 1). Comparison of HCC patients with liver cirrhosis cases with no diagnosis of HCC (n = 55) revealed significantly high AFP (p < 0.001) and IL-6 levels (p < 0.001) in the HCC group (Table 2 and Graph 1). A positive correlation of 61.2% was found between IL-6 and AFP, and this correlation was statistically significant (p < 0.001).

### Diagnostic Value of AFP and IL-6 in HCC Diagnosis

In the current trial, the AFP cutoff value for HCC was calculated as 5.93 [AUC 0.913, SD 0.026, p = 0.000, 95% confidence interval (CI) (0.863-0.963)]. The diagnostic value of AFP was separately calculated when compared with cutoff value of our laboratory of 13, and the cutoff values described in various trials were 20, 100, 200, and 400 (Table 3).

The cutoff value for IL-6 was calculated as 5.73 (pg/mL) [AUC 0.827, SD 0.036, p = 0.000, 95% CI (0.756-0.898)].

**Graphs 2A and B:**
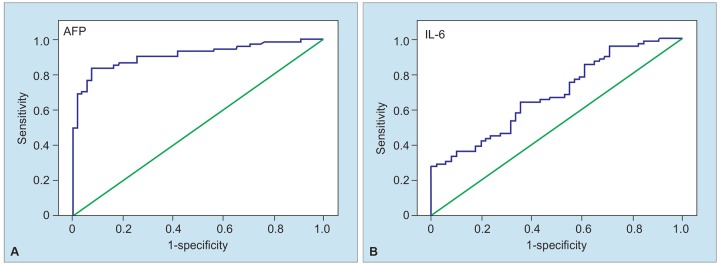
Diagnostic values of AFP and IL-6 in HCC (ROC curves)

The diagnostic value of this cutoff value is shown in Table 4 and Graph 2. The combination of AFP and IL-6 in diagnosis of HCC is shown in Table 4.

### Correlation of Tumor Markers with Child Score, Etiology of Cirrhosis, TNM Classification, Extrahepatic Spread and Portal Vein Invasion in Cirrhosis Patients (with and without HCC)

No difference was determined in the AFP (p = 0.396) and IL-6 (p = 0.126) levels in terms of Child score and etiology of cirrhosis. No correlation was detected between AFP and TNM classifications. In terms of IL-6, a significant difference was found between stage I, II and stage IV. No correlation was determined between levels of AFP and IL-6 and extrahepatic spread. Alpha-fetoprotein and plasma IL-6 levels were found to be correlated with portal vein invasion (Table 5).

**Table 1:** **Table 1:** Basal demographic, clinical, and laboratory characteristics of patients

		*Control*		*Cirrhosis*		*HCC*		*p-value (HCC/ Control)*		*p-value (HCC/ Cirrhosis)*	
n		30		55		75					
Age (mean ± SD)		65.20 ± 12.1		57.2 ± 12.7		62.8 ± 9.9		0.076		0.016	
Gender		18e, 12k		33e, 22k		54e, 21k		0.231		0.151	
AST (mean ± SD)		25.3 ± 12.8		63.8 ± 80.0		99.9 ± 138.5		0		0.016	
ALT (mean ± SD)		25.3 ± 16.9		41.6 ± 62.2		56.7 ± 114.0		0		0.076	
Total bilirubin (mean ± SD)		0.4 ± 0.2		3.7 ± 6.5		2.8 ± 4.4		0		0.171	
Direct bilirubin (mean ± SD)		0.16 ± 0.13		2.3 ± 5.4		1.8 ± 3.7		0		0.411	
Creatinine (mean ± SD)		–		1.01 ± 0.4		1.12 ± 1.20		–		0.947	
Albumin (mean ± SD)		–		3.07 ± 0.5		3.1 ± 0.6		–		0.386	
INR (mean ± SD)		–		1.4 ± 0.4		1.3 ± 0.2		–		0.141	
Child score		–		±		±		–		0.022	

**Table 2:** **Table 2:** Alpha-fetoprotein and plasma IL-6 levels in control group, cirrhotic patients, and HCC patients

		*Alpha-fetoprotein*				*Interleukin-6*			
		*Mean ± SD*		*Median (Min-Max)*		*Mean± SD*		*Median (Min-Max)*	
Control		1.98 ± 1.42		1.37 (0.61-6.89)		1.73 ± 1.29		1.69 (0.04-4.92)	
HCC		5,205.92 ± 19,598.68		6.88 (0.61-121000)		11.83 ± 16.51		5.06 (0.16-78.50)	
Cirrhosis		4.41 ± 11.46		2.32 (0.61-85.24)		3.03 ± 2.66		2.16 (0.16-10.80)	
HCC subgroup 1 (early stage)		15,422.74 ± 34,656.15		21.71 (1.12-121000)		12.80 ± 12.96		6.86 (0.70-40.30)	
HCC subgroup 2		1,215.17 ± 3,402.49		53.19 (2.50-13525)		17.19 ± 20.39		9.10 (0.80-78.50)	
HCC subgroup 3		10,110.36 ± 26,327.84		192.80 (0.80-121000)		19.93 ± 20.32		12.60 (0.50-78.50)	

**Table 3:** **Table 3:** Diagnostic value of AFP for HCC with six different cutoff values

		*Cutoff: 5.93*		*Cutoff: 13*		*Cutoff: 20*		*Cutoff: 100*		*Cutoff: 200*		*Cutoff: 400*	
Sensitivity		0.838		0.703		0.622		0.486		0.419		0.351	
Specificity		0.927		0.945		0.982		1.000		1.000		1.000	
PPV		0.939		0.945		0.979		1.000		1.000		1.000	
NPV		0.810		0.703		0.659		0.591		0.561		0.534	
Accuracy		0.876		0.806		0.775		0.705		0.667		0.628	

**Table 4:** **Table 4:** Interleukin-6 and combination of AFP with IL-6 (cutoff: 5.73) in diagnosis of HCC

		*IL-6: cutoff: 5.73*		*IL-6: cutoff: 5.73 + AFP cutoff: 5.93*		*IL-6: cutoff: 5.73 + AFP cutoff: 13*		*IL-6: cutoff: 5.73 + AFP cutoff: 20*		*IL-6: cutoff: 5.73 + AFP cutoff: 100*		*IL-6: cutoff: 5.73 + AFP cutoff: 200*		*IL-6: cutoff: 5.73 + AFP cutoff: 400*	
Sensitivity		0.707		0.892		0.811		0.770		0.730		0.730		0.730	
Specificity		0.885		0.808		0.827		0.865		0.885		0.885		0.885	
PPV		0.898		0.868		0.870		0.891		0.900		0.900		0.900	
NPV		0.676		0.840		0.754		0.726		0.697		0.697		0.697	
Accuracy		0.780		0.857		0.817		0.810		0.794		0.794		0.794	
Youden index		0.592		0.765		0.648		0.604		0.486		0.419		0.351	

**Table 5:** **Table 5:** Correlation of AFP and IL-6 levels in a total of 130 liver cirrhosis patients (55 with HCC, 75 w/o HCC) with Child scores, etiology of cirrhosis, TNM classification, extrahepatic spread, and portal vein invasion

				*AFP (median range)*		*IL-6 (median range)*		*p-value (AFP)*		*p-value (IL-6)*	
Child score		Child A		15.8 (0.6-1,200)		5.3 (0.16-78.50)		0.191		0.643	
		Child B		8.3 (0.8-12,100)		4.2 (0.3-78)					
		Child C		3.1 (0.6-12,100)		6.06 (0.6-78)					
Cirrhosis etiology		HBV (n = 57)		15.1 (0.6-12,100)		5.7 (0.3-78.5)		0.396		0.126	
		HCV (n = 29)		13.6 (0.7-3,674)		8.7 (0.8-41)					
		HBV + HCV (n = 3)		4.3 (0.7-40)		2 (0.4-12)					
		Delta (n = 8)		10.3 (1.1-1,421)		4.3 (0.6-78)					
		OIH (n = 2)		1.2		1.22					
		Wilson (n = 1)		2.28		1.01					
		Kriptojenik (n = 23)		3.04 (0.6-12,100)		3.1 (0.3-78.5)					
		Alkol (n = 7)		3.4 (1.8-5.6)		2.2 (0.8-6.2)					
TNM		Evre I (n = 3)		14.91 (1.1-18.7)		4.2 (1.2-4.6)		0.278		0.011	
		Evre II (n = 20)		40.8		(3.4-11,798)		9.8 (0.8-78.5)			
		Evre III (n = 14)		57.5		(2.5-31,928)		7.9 (0.9-25.9)			
		Evre IV (n = 38)		281.9 (80-12,100)		17.7 (0.8-78.5)					
Extrahepatic spread		Yes (n = 12)		40.5 (0.8-13,280)		19.9 (1-48.5)		0.164		0.312	
		No (n = 63)		102.8 (1-12,100)		10.8 (0.8-78.5)					
Prediction of portal vein invasion		Yes (n = 23)		111 (1.7-12,100)		21 (0.5-78.5)		0.022		0.09	
		No (n = 52)		34.5 (0.8-6,050)		9.8 (0.8-78.5)					

**Graphs 3A and B:**
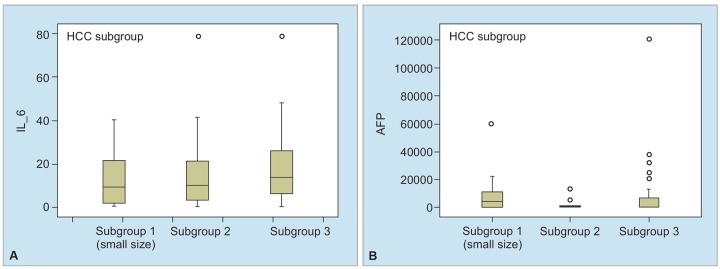
Box-plot graphs of AFP and IL-6 in HCC patient subgroups

### Comparison of HCC Subgroups in Terms of AFP and Plasma IL-6 Levels

Patients were classified in three subgroups in accordance with tumor diameter. Alpha-fetoprotein and IL-6 levels were compared in subgroups and effectiveness of these markers in subgroup 1 (tumor with small diameter) was investigated (Table 2 and Graph 3). Distribution of HCC patients in subgroups was as follows: 14 patients in subgroup 1, 18 patients in subgroup 2, and 43 patients in subgroup 3. No difference was detected in AFP (p = 0.600) and IL-6 (0.344) in all three subgroups. Alpha-fetoprotein and IL-6 provided no advantage in terms of early diagnosis of HCC. Comparison of each of these three HCC subgroups with cirrhotic patients revealed significantly higher AFP (p = 0.000) and IL-6 (p = 0.000) levels in each subgroup, as compared with cirrhosis cases (Table 2).

### Correlation of AFP and Plasma IL-6 Levels with HCC Survival

**Graphs 4A and B:**
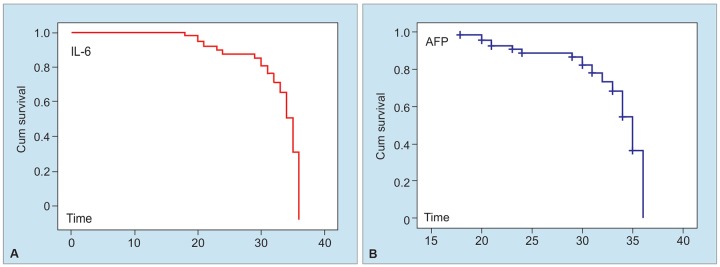
Survival graphs of AFP and IL-6

Correlation of AFP and plasma IL-6 levels with HCC survival was investigated by Cox regression and Kaplan-Meier curve. A total of 17 patients died during a mean follow-up period of 32.9 months [SD 0.668, SD 0.809, 95% CI (31.6-34.27)]. No correlation was found between mean AFP values and survival [p = 0.970, heart rate (HR) = 1.0, 95% CI (1.0-1.0)] (Graph 4). Likewise, mean IL-6 values and survival rates were found to be uncorrelated [p = 0.576, HR = 1.007, 95% CI (0.982-1.034)] (Graph 4).

## DISCUSSION

Hepatocellular carcinoma prognosis is strongly correlated with delay in diagnosis. Currently, diagnosis is finalized via imaging techniques and detection of high AFP levels.^11^ All of these methods are especially sensitive in diagnosis of large tumors. Therefore, novel markers for early and accurate diagnosis of HCC are closely associated with patient survival and public health. The most common cause of HCC in developing countries is hepatitis B virus (HBV), while in developed countries HCC is mainly associated with hepatitis C virus (HCV) and alcohol-induced liver cirrhosis.^12^ The most common cause among our patients was HBV infection, with a rate of 43.8%. In 55 of 130 patients with liver cirrhosis who were evaluated in the current trial, HCC was not diagnosed. Hepatocellular carcinoma was determined in 75 patients. A comparison of HCC patients with the control group revealed no difference between the two groups in terms of age and gender. The HCC patients were older than the cases in the cirrhosis group.

Alpha-fetoprotein is still the most frequently used marker in the diagnosis of HCC. In clinical trials, the most common cutoff value is 20 ng/mL. In trials where the AFP cutoff value was accepted as >20 ng/mL, sensitivity was calculated as 40 to 65%, specificity as 80 to 94%, the positive likelihood ratio as 3.1-6.8, and negative likelihood ratio as 0.4-0.6.^6^ Sensitivity and specificity of AFP in terms of different cutoff values were reported as follows :^13^ Alpha-fetoprotein cutoff 20 ng/mL (sensitivity 60%, specificity 91%), AFP cutoff 100 ng/mL (sensitivity 31%, specificity 99%), and AFP cutoff 200 ng/ mL (sensitivity 22%, specificity 99%).^13^

In the generation of tumor cells, angiogenesis, and inflammation, IL-6 is active. It activates JAK/STAT-3 (Janus kinase/signal transducer and transcription activator), MAPK (Ras/mitogen activated protein kinase), PI3K (phosphoinositol-3 kinase), and PkB/Akt (protein kinase B/Akt) pathways. Novel drugs acting on these signal pathways via IL-6 inhibition are being developed for cases with bone metastasis.^14^ In malignancies, elevation of IL-6 levels, a circular cytokine was shown in a number of studies. The role of IL-6 in carcinogenesis was indicated in various hematological and solid tumors.^15-18^ The role of pleotropic cytokine, IL-6, on liver damage and carcinogenesis was demonstrated.^7^ Cirrhotic patients are exposed to actions of various cytokines and especially to IL-6. Hepatitis B X-protein regulates IL-6 levels by NF-kB.^19^ Interleukin-6 acts as a mutagenic, motogenic, morphogenic, proneoangiogenic, and hepatocyte growth factor in HCC and thus plays a role in HCC carcinogenesis.

In addition, IL-6 takes part in carcinogenesis by acting on STAT-3 pathway. Interleukin-6 also decreases apoptosis in rat models. It was shown to decrease Fas-associated apoptosis.^7,20,21^

In chronic hepatitis B patients who were enrolled in this trial during 1997 to 2000 and followed up until 2008, the role of 27 cytokines on development of HCC was investigated. In 37 patients who developed HCC during follow-up period, serum IL-6 levels were determined to be higher than the remaining patients. Provided that a cutoff value of 7 pg/mL was accepted for IL-6 level, sensitivity was determined as 70%, specificity as 73%, PPV as 72%, and NPV as 71%. In the current trial, combining serum IL-6 level with AFP was shown to increase the diagnostic value for HCC.^22^ In a trial conducted on 29 healthy individuals, 50 chronic hepatitis patients due to HBV and HCV, 23 patients with cirrhosis, and 26 HCC cases, IL-6 was not found to be >3 pg/mL in any of the control cases. In the current trial, IL-6 level was determined to be high in HCC cases with an AFP of 20 ng/mL. Results of this trial enabled us to suggest that IL-6 may be beneficial in early diagnosis of HCC.^22^ Malaguarnera et al conducted a trial on 39 HCC patients and 25 healthy controls and showed that increased serum IL-6 levels were significantly higher in HCC cases, as compared with the control group. In addition, a positive correlation was reported for IL-6 levels and tumor size.^7^ In another trial, 30 HCC cases, 30 patients with liver cirrhosis, and 30 control cases were evaluated. Serum IL-6 levels were reported to be 4x higher in HCC patients, as compared with cirrhotic cases and 25x higher as compared with the control group (p < 0.0001). In addition, IL-6 levels were reported to be significantly higher among advanced HCC cases (Cancer of the Liver Italian Program score >3) (p = 0.003). In this trial, optimal IL-6 cutoff value for HCC with ROC curve was calculated as 7.9 pg/mL (sensitivity = 0.83, specificity = 0.83, effectiveness = 0.83). In addition, A_1_FP and IL-6 were combined by discriminant analysis in this trial and the end result for effectiveness of the combination of these two tests was reported as 82%.^21^

Thirty control cases, 55 liver cirrhosis patients without HCC, and 75 liver cirrhosis cases with HCC were evaluated in the current trial; AFP (p < 0.001) and IL-6 (p < 0.001) were found to be significantly higher among HCC cases, as compared with control group. Comparison of liver cirrhosis patients with and without HCC (n = 55) revealed significantly high AFP (p < 0.001) and IL-6 (p < 0.001) levels. The highest diagnostic value of AFP in our trial was determined with a cutoff value of 5.93. This cutoff value yielded a sensitivity of 83.8%, specificity of 92.7%, PPV of 93.9%, NPV of 81.0%, and an accuracy ratio of 87.6%. Results obtained with the cutoff value of our laboratory, namely 13, were as follows: sensitivity 70.3%, specificity 94.5%, PPV 94.5%, NPV 70%, and accuracy ratio 80.6%. Cutoff value for IL-6 was calculated as 5.73. Diagnostic values of this cutoff value were as follows: sensitivity 70.7%, specificity 88.5%, PPV 89.8%, NPV 67.6%, and accuracy ratio 78%. Combining the cutoff value of our laboratory for AFP (13) with the cutoff value for IL-6 (5.73) revealed the following diagnostic values: sensitivity 81.1%, specificity 82.7%, PPV 87%, NPV 75.4%, and accuracy ratio 81.7%.

Alpha-fetoprotein and IL-6 were compared in subgroups and the effectiveness of these markers was investigated in subgroup 1 (small-sized tumor). No significant difference was found between the three subgroups in terms of AFP (p = 0.600) and IL-6 (0.344).

Alpha-fetoprotein and IL-6 provided no advantage in early diagnosis of HCC. The correlation of AFP and plasma IL-6 levels with HCC survival was examined by Cox regression and Kaplan-Meier curve. Seventeen patients died during a mean of 32.9 months. Mean AFP and IL-6 levels were regarded to have no correlation with survival. No correlation was found between AFP and IL-6 levels with the etiology of cirrhosis, child scores, TNM classification, and extrahepatic spread. Alpha-fetoprotein and plasma IL-6 levels were found to be correlated with portal vein invasion.

In conclusion, plasma IL-6 level was found to be significant in the diagnosis of HCC. However, the significance of IL-6 in the diagnosis of HCC was not high, as compared with AFP. The combination of plasma IL-6 level and AFP did not lead to an increase in diagnostic value. Alpha-fetoprotein and IL-6 provided no advantage in terms of early diagnosis of HCC and no correlation was observed between these markers and survival.
